# Non-alcoholic Fatty Liver Disease and Gallstones: A Systematic Review

**DOI:** 10.7759/cureus.45027

**Published:** 2023-09-11

**Authors:** Ethan Slouha, Stefan J Biput, Azeez 0 Kuteyi, Amy E Kalloo, Vasavi Rakesh Gorantla

**Affiliations:** 1 Medical School, St. George's University School of Medicine, True Blue, GRD; 2 Clinical Sciences, St. George's University, True Blue, GRD; 3 Anatomical Sciences, St. George's University, True Blue, GRD

**Keywords:** bi-directional, triglycerides, total cholesterol level, non alcoholic fatty liver, gallstones

## Abstract

Non-alcoholic fatty liver disease (NAFLD) is steatosis of the liver that resembles alcohol-induced liver injury but is a metabolic disorder. Most patients are obese with increased triglyceride levels due to increased intake of fatty food, which can cause excess fat to build up in the liver. At the same time, continuous ingestion of fatty foods can lead to gallstones (GS) due to the overproduction of cholesterol. NAFLD and GS have been seen to coincide, and there might be a relationship between them. This systematic review analyzes the incidence of NAFLD and GS to determine a bidirectional relationship. A comprehensive literature review was done using ProQuest, PubMed, and ScienceDirect, and included only experimental studies and meta-analyses. The search included the keywords ‘gallstones and non-alcoholic fatty liver disease’ and ‘cholelithiasis and non-alcoholic fatty liver disease’. Our initial search included 10,665 articles and was narrowed down to 19 through extensive inclusion and exclusion criteria. There is a bidirectional relationship between the incidence of NAFLD and GS, where an increase in either can lead to an increase in the other. Both NAFLD and GS share similar risk factors leading to the development of each disease. On average, there’s an increase in the prevalence of gallstones in NAFLD patients, and patients with GS were also more likely to have NAFLD. There was a prevalence of NAFLD in those with asymptomatic gallstones as well, indicating that the risk factors are crucial in the development of both. As a result, some research is determining whether an evaluation of the liver should be routine during cholecystectomy due to the increased risk of developing NAFLD.

## Introduction and background

Non-alcoholic fatty liver disease

Nonalcoholic fatty liver disease (NAFLD) is a condition diagnosed with liver imaging, specifically ultrasound, when there is an accumulation of fat, a disorder known as steatosis. This fat accumulation makes up more than 5% of the weight of the liver, in the absence of alcohol as the cause but resembles alcohol-induced liver injury [1]. NAFLD is categorized as a metabolic disorder due to the role of hormones, nutrition, and genetics in its pathogenesis [2]. Most patients with NAFLD are obese and therefore have an increased serum triglyceride level. As in obesity, there is an increased intake of fatty foods with reduced energy output [3]. Furthermore, in patients with insulin resistance, there is decreased storage of lipids in adipose tissue, which leads to increased lipolysis, allowing excess free fatty acids to enter hepatocytes resulting in steatosis; additionally, the free fatty acids in the liver undergo oxidation leading to further liver damage [1]. Therefore, based on the study by Pouwels et al., the first step of acquiring NALFD is the accumulation of lipids in hepatocytes which eventually leads to insulin resistance, leading to the second step, which is oxidative stress resulting in further liver damage and inflammation [2].

Gallstones

Gallstones (GS) are precipitations in the gallbladder, an organ located beneath the liver that stores, concentrates, and releases bile into the small intestines [4]. GS are comprised of cholesterol, bilirubin, and calcium salts with some proteins [5]. GS are normally asymptomatic, but symptoms arise when the stone obstructs the biliary passage leading to abdominal pain called biliary colic [4]. Most GS are cholesterol GS, but there are also black-pigmented GS and brown-pigmented GS which primarily consist of calcium hydrogen bilirubinate [5]. Cholesterol GS are created when there is excessive secretion of cholesterol into bile by hepatocytes or impaired emptying or hypomotility of the gallbladder [6]. Excess cholesterol cannot dissolve in the bile and precipitates as crystals that eventually form stones [5]. Furthermore, if the gallbladder has impaired motility and cannot empty the bile, the bile becomes concentrated and begins to harden to form stones [6]. The aim of this paper is to assess the relationship and prevalence of GS with NAFLD due to their similarities in pathogenesis.

Methods

An exhaustive and extensive literature search was done using PubMed, ProQuest, and ScienceDirect databases from January 1st, 2002, to December 31st, 2022. Keywords included ‘gallstones and nonalcoholic fatty liver disease’ and ‘cholelithiasis and nonalcoholic fatty liver disease’. The electronic search focused on peer-reviewed, experiment publications that were in line with the scope of this paper. Publications not written in English, published prior to 2002 and duplicates were excluded from the screening process. Once the publications were found, three independent co-authors examined the information. The publications found in the search were examined based on their full-text accessibility, study type, title, and abstracts. The initial search of the three databases resulted in 10,665 publications. The selected publications were further narrowed down based on keyword specifics and the overview provided by the abstracts. A total of 19 publications were found to be within scope, according to the following criteria.

Inclusion Criteria

The following inclusion criteria were used: publications written in English, conducted on humans, published between 2002 and 2022, focused on the prevalence and incidence of gallstones and NAFLD together, full-text inclusive of both subscription and non-subscription articles, and peer-reviewed with variations between cohort, case-control, observation studies, and meta-analyses.

Exclusion Criteria

**Figure 1:**
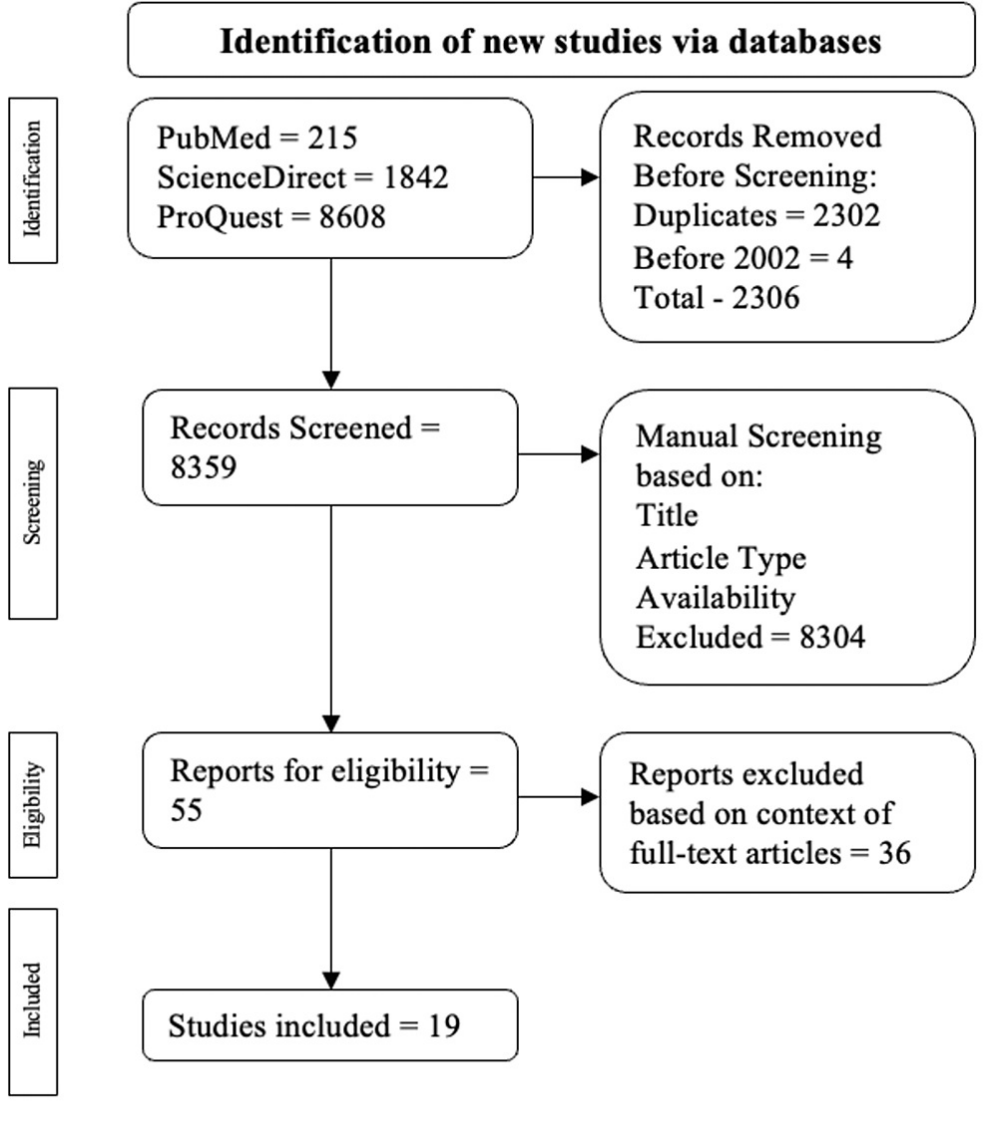
Algorithm used to filter articles based on the study's inclusion and exclusion criteria

Exclusion criteria included case series/reports, systematic reviews, and review articles. All non-full-text publications and duplicates were also excluded. The procedure of inclusion and exclusion of the publications is drawn out in Figure 1.

Bias

All studies were assessed for bias. The studies showed a medium risk of bias as most studies disclosed their protocols and methods and focused on databases. The individual risk of bias was evaluated using the Grading of Recommendation, Assessment, Development, and Evaluations (GRADE) tool, which assessed flaws like imprecision, indirectness, and publications.

The algorithm for this literature review was done as described in the PRISMA statement [7].

## Review

Results

A total of, 10665 publications were found; 215 were from PubMed, 1842 were from ScienceDirect, and 8608 were from ProQuest. Among the exclusions, 2302 were duplicate publications, and four were published before 2002. This resulted in 2306 publications being excluded during the automatic screening process, leading to 8359 publications for manual screening. Publications were manually screened based on title, study type, abstract, and availability, resulting in 55 articles being checked for eligibility. Ultimately 19 articles were used.

Due to the similarities in pathogenesis, there should be a corresponding incidence rate between GS and NAFLD. It was found that there is a bidirectional relationship between GS and NAFLD with a significant association. Biomarkers of NAFLD include macroglobulins, haptoglobulins, total bilirubins, transferases, and transaminases. A single marker alone of NAFLD increases the chance of developing GS. Elderly individuals and females are more likely to develop GS with an underlying NAFLD diagnosis. Research is unsure whether the trend is the same with asymptomatic gallstones. There is also a greater risk of NAFLD for those who’ve undergone a cholecystectomy.

Discussion

There is a bidirectional relationship between the prevalence of NAFLD and gallstones, indicating an increased risk in both [8]. There is a positive correlation between NAFLD and GS in that the risk factors for developing both diseases were similar [9, 10]. There is a significant association between NAFLD and GS, even when accounting for heterogeneity [11, 12]. The presence of gallstones and previous cholecystectomy increased significantly in NAFLD patients [13]. Koller et al. observed that 19% of patients have both gallstones and one marker of NALFD [8]. A patient who has at least one marker of fatty liver disease, whether it is an increase in macroglobulins, haptoglobulins, total bilirubins, transferases, or aminotransaminases, had nearly a double prevalence of gallstones compared to patients with no markers (p < 0.0001) [8]. The development of GD was positively correlated with the grade of NAFLD (p < 0.001) [14, 15].

Prevalence of Gallstones and NAFLD

The prevalence of GS in NAFLD varied between studies but averaged about 15% [10, 14, 16, 17]. One of the mechanisms underlying this occurrence may be due to NAFLD leading to excessive cholesterol accumulation and altered cholesterol metabolism resulting in the development of GS [18]. Patients with GS were more likely to have NAFLD by 6.85 times (p < 0.01) compared to those with cholecystectomy, which were 2.14 times more likely [19]. Subjects with GS had higher rates of metabolic syndrome, hypertension, medication use for diabetes, dyslipidemia, and high-grade NAFLD than those without [15]. GS was independently associated with NAFLD when accounting for age, sex, and metabolic profile [13]. Ahmed et al. did observe that those who had a family history of GS and those who had GS with NAFLD had a first-degree relative with GS (p < 0.034) [20].

Kichloo et al. observed that Caucasian patients with NAFLD had a higher prevalence of GS which was not replicable in other racial groups [19]. GS was significantly associated with older patients, and this trend continued with those who also had NAFLD [10, 13, 17, 19, 21, 22]. This may be due to reduced serum high-density lipoprotein (HDL) as HDL is needed to regulate cholesterol secretion (Hung et al., 2020). Female patients with NAFLD were also more likely to develop GS complications with an increased prevalence [11, 14, 17, 19, 23]. However, one study found no statistically significant association with the development of GS [18].

Prevalence of Asymptomatic Gallstones and NAFLD

There is debatable research on whether asymptomatic GS are associated with NAFLD or not, as most people are unaware that they have GS. The prevalence of NAFLD was 59% in patients with asymptomatic gallstones compared to 46.7% in those without gallstones [21, 24]. A significant association exists between NAFLD and increased incidence of asymptomatic GS after accounting for metabolic risk factors (p = 0.006) [24]. This association is stronger in females and patients who were < 50 years of age (p < 0.0001 for both) [24]. However, one study found that asymptomatic GS was negatively associated with NAFLD** **[25].

Prevalence of Cholecystectomy and NAFLD

Several studies evaluated the relationship between cholecystectomy and NAFLD and whether routine liver analysis should be standard for these patients. Hajong found that of 200 patients undergoing cholecystectomy, 138 had negative testing for NAFLD, 39 were borderline or inconclusive, and 23 had definitive tests [26]. Patients who had cholecystectomy had a 35% increase in the risk of developing NAFLD (Kwak et al. [13]). Vice versa, cholecystectomy's prevalence was significantly higher in patients with NAFLD [25]. Additionally, there was a significantly stronger association between NAFLD and the incidence of cholecystectomy in women than in men (p < 0.033) [18]. The increased risk of developing NAFLD may be because cholecystectomy leads to decreased fibroblast growth factor 19, which affects cholesterol metabolism, causing triglycerides to accumulate in the liver [18]. The grounds for routine liver biopsy during or after cholecystectomy for GS are substantiated due to the high occurrence of NAFLD in these patients, which can escape detection for an extended period [9].

Some limitations of this article arose because of the limited number of studies analyzing the relationship between GS and NAFLD. There’s research about the varying pathogenesis that may correlate to a relationship, but these are the only articles that address this relationship. This article also excluded animal studies that could elucidate the actual relationship displayed. A summary of the discussion/reviewed findings is presented in Table 1. 

**Table 1:** Summary of discussion findings NAFLD: non-alcoholic fatty liver disease, NASH: non-alcoholic steatohepatitis, BMI: body mass index, DM: diabetes mellitus, CI: confidence interval, ALT: alanine transaminase, HOMA: homeostatic model assessment (quantifies insulin resistance), LDL: low-density lipoprotein, ALP: alkaline phosphatase, HDL: high-density lipoprotein, FPG: fasting plasma glucose, OR: odds ratio, GS: gallstones, USG: ultrasonogram, GD: gallstone disease.

	Author	Country	Design & Study Population	Findings	Conclusion
1	Ahmed et al., 2017 [20]	Pakistan	Retrospective study (n = 90)	55 persons (62.5%) had NAFLD, and 25 persons (28.4%) had metabolic syndrome, both groups having gallstones. Those with NAFLD had a significant p-value of 0.034 with their first-degree relative having cholelithiasis whereas those with metabolic syndrome had a non-significant p-value of 0.190.	Metabolic syndrome is associated with gallstones and NAFLD, where patients whose first-degree relatives had gallstones represented a large percent of those with gallstones and was highly evident for patients who also had NAFLD.
2	Alsaif et al., 2020 [21]	Saudi Arabia	Cross-Sectional Study (n = 301)	301 patients with gallstones were selected of which 143 (47.8%) had NAFLD, 125 (41.8%) had steatosis, and 18 (6%) had NASH. It was found that NAFLD was strongly associated with the patient’s age (r=0.243, p<0.0001) and BMI (r=0.242, p<0.0001). Of the 301 patients with gallstones, 15% had a normal BMI, 29% were overweight, and 56% were obese. Those with a BMI of 30-40 had a greater risk of NAFLD by 6.145 (p=0.002) which further increased to a risk of 6.145 (p=0.002) in those with a normal BMI. Patients presenting with gallstones and type 2 DM had an increased risk of NAFLD by 2.839 times (p=0.015).	47.8% of persons with gallstones had associated NAFLD, with 41.8% having steatosis and 6% with NASH. Transient elastography (FibroScan) can be a beneficial screening tool due to its accuracy in measuring the levels of liver steatosis and fibrosis in NAFLD. Due to the occurrence of NAFLD in patients with gallstones, it will be beneficial to select patients if a routine liver biopsy is performed simultaneously during cholecystectomy.
3	Chang et al., 2018 [18]	Seoul, Korea	Longitudinal cohort study (n = 283,446; n = 219,641)	Of the 283,446 patients without gallstones/cholecystectomy initially, 6,440 eventually developed gallstones. Of the 219,641 patients without NAFLD initially, 49,301 developed NAFLD. The hazard ratio was 1.26 (95% CI=1.17-1.35) for the development of gallstones in the NAFLD group compared to the non-NAFLD group. The hazard ratio was 1.14 (95% CI=1.07-1.22) and 1.17 (95% CI=1.03-1.33) for development of NAFLD in the gallstone/cholecystectomy sample compared to the no gallstone sample. Non-invasive fibrosis markers were grade- and dose-dependent in the incidence of gallstone (p=<0.01).	NAFLD and non-invasive fibrosis markers were not associated with the risk of developing gallstones, however, gallstones and cholecystectomy did play a role in the incidence of NAFLD. Further analysis is required to understand the causation between gallstones and cholecystectomy with NAFLD.
4	Fracanzani et al., 2012 [14]	Italy	Cross-sectional study (n = 524)	524 patients (373 males) were selected, and gallstones were diagnosed in 20% (108) and NASH was diagnosed in 60% (313) via liver biopsy. The diagnosis of gallstones increased with increasing fibrosis (p=0.0001) and with severity of necrosis and inflammation (p=0.01). OR=1.37(95%CI=1.04-1.8) for gallstones and female gender. OR=1.027(95%CI=1.003-1.05) for gallstones and age. OR=1.21 (95%CI=1.10-1.33) for gallstones and fasting glucose. OR=1.40(95%CI=1.06-1.89) for gallstones and NASH. ALT levels had a lower risk of gallstones as the OR=0.98(95%CI=0.97-0.99).	It is useful to do a routine liver biopsy for an early diagnosis during cholecystectomy as 50% of patients with gallstones had NAFLD.
5	Garcia-Monzon et al., 2015 [10]	Spain	Prospective cohort study (n = 215)	215 patients with gallstones referred for cholecystectomy underwent abdominal ultrasound and liver biopsy of which 10.2% had NASH, and 41.4% had simple steatosis. Of the patients with gallstones and NASH, there was a higher HOMA (homeostatic model assessment) score than patients with simple steatosis with a p-value of 0.015. Of the patients with gallstones and NASH, 90.9% had fatty liver on ultrasound compared to patients with NASH (61.8%) with a p-value of 0.044.	Increased HOMA score and fatty liver on ultrasound are accurate factors to predict NASH in patients with gallstones and as a result, patients with gallstones who have a history of insulin resistance and fatty liver should undergo needle liver biopsy during their cholecystectomy procedure.
6	Hajong et al., 2019 [26]	India	Cross-sectional study (n = 200)	A sample of 200 (140 females & 60 males) with gallstones participated in the study. 138 had non-alcoholic steatohepatitis (NASH), 39 had borderline/suspicious NASH, and 23 had definitive NASH. In those patients with NASH, they had increased BMI, weight, total cholesterol, LDL, ALP, and weight circumference.	31% of patients with cholelithiasis had NAFLD leading to patient education about lifestyle changes regards to exercise and diet, as well as the progressiveness of NAFLD to cirrhosis or hepatocellular carcinoma. Due to the high prevalence, it is recommended to perform a routine liver biopsy during cholecystectomy for early diagnosis and treatment.
7	Hung et al., 2020 [22]	Taiwan	Cross-sectional study (n = 3,037)	Of the sample of 3,037 patients, the mean age was 73.6 years (std=6.0), over 70% were obese, and 17.7% had gallstones. Metabolic syndrome had a positive relationship with gallstones with an odd ratio of 1.31 (95%CI=1.05-1.64) with a p-value of 0.020. Factors of metabolic syndrome such as low HDL (OR=1.35, 95%CI=1.10-1.64, p-value of p<0.001), and increased fasting plasma glucose (OR=1.36, 95%CI=1.10-1.69, p-value of p<0.001) increased the likelihood for gallstone disease in NAFLD elderly patients.	For elderly patients with NAFLD, there is an increased risk of gallstone disease due to metabolic syndrome and factors such as reduced HDL levels and increased fasting blood glucose levels; for prevention of gallstone disease in NAFLD patients, their FPG and HDL levels must be monitored as well as educating the patient/s about symptoms of gallstones disease.
8	Jaruvongvanich et al., 2016 [12]	USA	Meta-analysis (n = 79,629)	Patients having both NAFLD and gallstones had a pooled OR of 1.55 (95%CI = 1.31-1.82) with the statistical between-studies heterogeneity being 64%. With only cohort studies, the association between NAFLD and gallstones was OR=1.33 (95% CI = 1.14-1/55) with the statistical between-studies heterogeneity being 0%, therefore the association remained significant.	Gallstone disease is significantly associated with NAFLD.
9	Ramos-De la Medina et al., 2008 [9]	Mexico	Cohort study (n = 95)	95 patients were evaluated. Patients were placed in two groups, Group A and Group B. 45% of the total number of subjects were placed in Group A and they had normal liver biopsies. 55% of the patients were in group B and showed signs of NAFLD. Comparisons between both groups showed that the subjects in Group B had higher BMIs and were older	Patients who exhibit risk factors for the development of NAFLD would benefit from a liver biopsy during cholecystectomy for GD.
10	Shen et al., 2017 [17]	China	Meta-Analysis (n = 45,004)	The pooled prevalence of GS with participants who had NAFLD was 17% (95% CI: 0.12-0.23). After evaluating the non-NAFLD group, NAFLD had a remarkable association with GS (OR:1.40, 95% CI: 1.36-2.79). Participants in the group with GS had more females, were older, and had higher BMIs in comparison to the non-GS group.	When compared to the general population, people who have NAFLD tend to also present with GS. There is also a remarkable correlation between GS and the female gender, old age and increasing BMI.
11	Singh et al., 2019 [23]	India	Observational Study (n = 101)	101 participants were included in the study. The mean age was 42.36 (Range 18-70 years old). Seven patients had diabetes and 11 were hypertensive. 35 patients had NAFLD on USG. The mean age of the patients with NAFLD was 44.5 (Range: 21-67 years old). Three of the seven diabetic patients and four of 11 hypertensive patients had NAFLD on liver biopsy	GS and NAFLD have some of the same risk factors and patients that have GS have a high chance of NAFLD.
12	Yilmaz et al., 2014 [16]	Istanbul	Cross-sectional study (n = 441)	Of the sample of 441 patients, 54 patients (12.2%) had gallstone disease (GD). 77% of the patients that had GD had undergone cholecystectomy for symptomatic GD. The patients who were GD+ were found to be older, had higher BMIs, and were more likely to be female. It was also found that patients who had GD did not have a higher risk for fibrosis or non-alcoholic steatohepatitis on histology.	The study shows that the prevalence of GD has a positive relationship with age, female sex, obesity, and the metabolic syndrome but not with an increased risk of non-alcoholic steatohepatitis and significant fibrosis in patients with NAFLD
13	Kichloo et al., 2021 [19]	USA	Retrospective study (n = 14294784)	3.3% of patients with gallstone disease were found to also have NAFLD. Further, the prevalence of NAFLD in women with gallstone disease was 64.3% compared to men which was 35.7%. There is an association with gallstone disease and NAFLD and an association with NAFLD and cholecystectomy. NAFLD association with gallstones was stronger in men (OR = 6.67) than in women (OR = 6.05), but NAFLD association with cholecystectomy was stronger with women (OR = 2.01 vs. OR = 1.85) (p < 0.01 for all).	NAFLD has a higher prevalence in women with gallstone disease in women than men, and there maybe a risk in NAFLD due to cholecystectomy/gallstones.
14	Kim et al., 2019 [15]	Korea	Cohort study (n = 7886)	Higher grade of NAFLD and older age were independent risk factors for gallstones. There is a strong correlation between gallstone disease and the grade of NAFLD on ultrasound	NAFLD based on grade is associated with gallstone disease.
15	Koller et al., 2012 [8]	Slovakia	Retrospective study (n = 482)	There was a significantly higher prevalence of gallstones among patients with NAFLD compared to those without (p < 0.0001). Gallstones are independent predictors of NAFLD (OR = 1.77). 56% of patients with gallstones had NAFLD compared to 33% who did not. NAFLD is also an independent predictor of gallstones (OR = 1.92). Metabolic risk factors such as BMI, triglycerides, diabetes, and total cholesterol concentrations were also independent predictors of NAFLD and gallstones	Gallstones are an independent risk factor of NAFLD and vice versa along with metabolic risk factors.
16	Kwak et al., 2015 [13]	Korea	Cross-sectional study (n = 17612)	The prevalence of gallstones, including a history of cholecystectomy, was increased significantly in NAFLD patients. Vice versa, there was an increase in the prevalence of NAFLD in patients with gallstone disease. While cholecystectomy is associated with NAFLD, gallstones were not (p = 0.028 and p = 0.153 respectively).	Gallstones are not independently associated with NAFLD, compared to cholecystectomy.
17	Liu et al., 2014 [11]	China	Longitudinal study (n = 498)	There is an association between gallstones and NAFLD (p = 0.047). After adjustment for sex, it was found that specifically in females there was an association between NAFLD and gallstones (p = 0.001).	NAFLD is associated with gallstones with a stronger association apparent in females compared to males.
18	Lu et al., 2021 [25]	China	Cross-sectional study (n = 4325)	There was no significant difference in the rate of gallstones between subjects with and without NAFLD before and after case-control matching (p = 0.15 for both). The prevalence of NAFLD in subjects with asymptomatic gallstones was reduced compared to those without gallstones. The prevalence of NAFLD was higher in patients who had undergone cholecystectomy. Symptomatic gallstones and cholecystectomy were strongly associated with NAFLD.	Due to higher proportion of cholecystectomy and lower amount of asymptomatic gallstones in subjects with NAFLD may indicate that NAFLD can increase the risk of complications of gallstones.
19	Qiao et al., 2017 [24]	China	Cross-sectional study (n = 7583)	Prevalence of NAFLA was significantly raised in patients with asymptomatic gallstones compared to those without asymptomatic gallstones (p < 0.0001). NAFLD associated with asymptomatic gallstones was higher in females and subjects < 50 years of age (p = 0.0009 and p < 0.0001 respectively).	Asymptomatic gallstones are strongly associated with non-alcoholic fatty liver disease.

## Conclusions

Based on this research, there is a bidirectional relationship between the development of GS and NAFLD. Patients with NAFLD have a significantly higher prevalence of GS throughout their disease. This correlation falls back to the pathogenesis of both NAFLD and GS, which have origins in dysfunctional cholesterol and fat metabolism. Patients more likely to develop GS concurrently tend to be Caucasian, elderly, and female. Going further into the association, we questioned whether this relationship occurred with patients suffering from asymptomatic GS. Research, however, is still split on whether there’s an association with NAFLD. There’s also a significant association between the incidence of cholecystectomy and NAFLD. The increased risk of developing NAFLD may be due to a decrease in fibroblast growth factor 19, another alteration to cholesterol metabolism. Further research should be conducted to determine if routine testing for the possible association should be done during a typical workup due to their hand-in-hand relationship.
